# The Fallacy of Using Administrative Data in Assessing the Effectiveness of Food Fortification. Comment on: “Folic Acid Fortification and Neural Tube Defect Risk: Analysis of the Food Fortification Initiative Dataset. *Nutrients* 2020, *12*, 247”

**DOI:** 10.3390/nu12051352

**Published:** 2020-05-08

**Authors:** Vijaya Kancherla, Helena Pachón, Hannah Blencowe, Homero Martinez, Godfrey P. Oakley, Robert J. Berry

**Affiliations:** 1 Department of Epidemiology, Emory University Rollins School of Public Health, Atlanta, GA 30322, USA; gpoakley@mindspring.com (G.P.O.J.); rjberry.atlanta@gmail.com (R.J.B.); 2 Hubert Department of Global Health, Emory University Rollins School of Public Health, Atlanta, GA 30322, USA; helena.pachon@emory.edu; 3 Food Fortification Initiative, Atlanta, GA 30322, USA; 4 Maternal Adolescent Reproductive and Child Health Centre, London School of Hygiene and Tropical Medicine, London WC1E 7HT, UK; hannah-jayne.blencowe@lshtm.ac.uk; 5 Nutrition International, Ottawa, ON K2P 2K3, Canada; hmartinez@NUTRITIONINTL.ORG

## Abstract

Our objective in this comment is to highlight several limitations in an ecological research study that was published in *Nutrients* by Murphy and Westmark (2020) in January 2020. The study used data from the Food Fortification Initiative (FFI) website, and applying an ecological study design, made an error of “ecologic fallacy” in concluding that “national fortification with folic acid is not associated with a significant decrease in the prevalence of neural tube defects (NTDs) at the population level”. We list study limitations that led to their erroneous conclusions, stemming from incorrect considerations regarding NTD prevalence, the average grain availability for a country, the fortification coverage in a country, the population reach of fortified foods within a country, and the absence of the consideration of fortification type (voluntary vs. mandatory), country-specific policies on elective terminations for NTD-affected pregnancies, stillbirth proportions among those with NTDs, and fortification implementation. FFI data are derived from many sources and intended for fortification advocacy, not for hypothesis testing. The flawed study by Murphy & Westmark (2020) in *Nutrients* promotes a confusing and incorrect message to stakeholders, misguides policy makers, and hinders progress in global NTD prevention through a cost-effective, safe, and effective intervention: the mandatory large-scale folic acid fortification of staple foods.

Murphy & Westmark (2020) [1], published in *Nutrients* in January this year, using data from the Food Fortification Initiative (FFI) website (www.ffinetwork.org) and an ecological study design, have made an error in their study conclusion. Their study conclusion contradicts several systematic reviews and meta-analyses published earlier [2,3,4,5]. Ecologic fallacy is an inherent error in ecological study designs that lack individual-level data and rely on aggregate information pooled nationally or sub-nationally, collected by non-standardized sources.

We list below major limitations in Murphy & Westmark’s study that have led to their erroneous conclusion:

1. The modeled prevalence estimates for neural tube defects (NTDs) [6] used in their analysis have inherent biases and limitations, and they underestimate the true prevalence of NTDs for many developing countries that lack birth defect surveillance. They are mainly intended to provide policy makers with a crude burden of NTDs and not for scientific hypothesis-oriented research.
2. The FFI’s individual country profiles that contain grain fortification-related information are intended for stakeholders in the flour and rice milling industry, and organizations and policy-makers invested in grain fortification. Their variable “Folic acid fortification measured in ppm” is an incomplete measure of fortification reach and impact. The average fortification levels are meaningless without integrating data regarding the average grain availability for a country [7]. A low fortification level in a country with high grain availability would have a very different fortification impact compared to a low fortification level in a country with low grain availability.
3. Fortification compliance in a country was not considered, which is available online at https://fortificationdata.org/map-proportion-of-food-vehicle-that-is-fortified/. Several countries have a mandatory fortification policy, but where less than 100% of the food is fortified.
4. The population reach of fortified foods, which indicates the actual consumption of fortified foods, was not considered. Analyzing the population average fortification levels (ppm), without considering the reach and coverage of the fortified product [7], masks the differences found between consumers and non-consumers.
5. Other review studies [3,5] showing the effectiveness of fortification on NTD prevention were not cited.
6. Several supporting factors, including fortification type (voluntary vs. mandatory), country-specific policies on elective terminations for NTD-affected pregnancies, stillbirth proportions among those with NTDs, and fortification implementation were not considered.

According to the Centers for Evidence-Based Medicine, in the hierarchy of scientific evidence, evidence generated by ecological studies provides the lowest level of significance in establishing causality [8]. The FFI data are derived from many sources and intended for fortification advocacy, not for hypothesis testing. The flawed study by Murphy & Westmark (2020) in *Nutrients* promotes a confusing and incorrect message to stakeholders, misguides policy makers, and hinders progress in global NTD prevention through a cost-effective [9], safe [10] and effective [2,3,4,5] intervention: the mandatory large-scale folic acid fortification of staple foods.
